# Magnitude and Reasons for Harmful Traditional Practices among Children Less Than 5 Years of Age in Axum Town, North Ethiopia, 2013

**DOI:** 10.1155/2014/169795

**Published:** 2014-06-19

**Authors:** Kahsu Gebrekirstos, Atsede Fantahun, Gerezgiher Buruh

**Affiliations:** Department of Nursing, College of Health Sciences, Mekelle University, Mekelle, 18713 Tigray, Ethiopia

## Abstract

*Background.* In addition to beneficial traditional practices, there are around 140 harmful traditional practices affecting mothers and children in almost all ethnic groups of Ethiopia. Therefore this study might give a clue about their practice and associated factors. The objective of this study was to assess magnitude of harmful traditional practices among children less than 5 years of age in Axum Town, North Ethiopia. *Methods.* Community based cross-sectional study was conducted on 752 participants who were selected using multistage sampling. Simple random sampling method was used to select ketenas from all kebelles of Axum Town. After proportional allocation of sample size to eachketena, systematic random sampling method was used to get the study participants. Data was collected using interviewer administered questionnaire; it was entered and analyzed using SPSS version 16 and descriptive statistics was calculated. *Results.* Majority of the respondents (81.2%) were Orthodox, 78.2% of the mothers had no work, and majority of mothers had no formal education. Among the harmful traditional practices performed on children, uvula cutting alone was performed on 72.8% of children followed by milk teeth extraction and uvula cutting with eyebrow incision. *Conclusion.* The leading harmful traditional practice performed on children in this study was uvula cutting.

## 1. Introduction

WHO defined traditional medicine in 1978 as “the sum total of all the knowledge and practices, whether explicable or not, used in diagnosis, prevention and elimination of physical, mental or social imbalance and relying exclusively on practical experience and observation handed down from generation to generation whether verbally or in writing” [1, 2]. Even though the prevalence and degree may vary, harmful traditional practices (HTPs) which have numerous long term devastating effects are also performed in all continents of the world [3]. United Nations (UN) agencies and human right bodies started addressing HTP in the early 1990s but there was little progress [4]. There are now a number of important international instruments endorsed by most of the governments and could serve as a basis for a struggle against HTPs [5]. In addition to deep-rooted beliefs, customs, and rational attitudes, lack of knowledge and being unaware of the effects of the practices help maintain these problems. HTPs such as uvulectomy, tonsillectomy, female circumcision, milk teeth extraction, and eyebrow incision are widely practiced with no or little attention to hygiene in Ethiopia [1]. Sometimes a harmful practice is so deeply rooted that it seems impossible to change. But in every country people have pushed forward positive social changes, and harmful practices have been ended [6]. Unlike in developing countries where traditional practices are performed by more than 80% of the population, populations in some countries in the Middle East as well as immigrants to Europe and USA have abandoned these practices [7].

Traditional medical and behavioral practices in sub-Saharan Africa have been evaluated infrequently in relation to risk of infectious disease transmission [8]. Kupeli et al. reported adverse effects and immediate, short-term, and long-term complications immediately after the procedure; the most common risks include excessive bleeding, infection, tetanus, meningitis, transmission of infectious diseases (HIV and hepatitis), and death [9].

It is said that there are around 140 HTPs affecting mothers and children in almost all ethnic groups of Ethiopia. HTPs that affect children are female genital mutilation (FGM), milk teeth extraction (MTE), food taboo, uvula cutting (UC), forbidding food and fluids during diarrhea, keeping babies from exposure to sun, and feeding new born babies with fresh butter [7].

In Ethiopia, two important national surveys have been conducted by EGLDAM (Ye Ethiopia Goji Lemadawi Dirgitoch Aswagaj Mahber) and the Former National Committee for Traditional Practices of Ethiopia (NCTPE). The survey identified five top priority HTPs including FGM, uvula cutting, MTE, early marriage, and marriage by abduction at national level [10]. Based on the baseline survey (BLS) on HTPs in Ethiopia conducted in 1997, the prevalence of uvula cutting in Tigray Region was 92.8% but 66.4% on the follow-up survey. The prevalence of FGM in Tigray Region was 48.1% in BLS with marked decrease to 21.2% in follow-up survey. The prevalence of MTE in Tigray Region decreased from 52.4% (baseline survey) to 26.6% (follow-up survey) [4].

The Objective of this study was to assess the magnitude and reasons associated with HTPs among Children less than 5 years of age in Axum Town, North Ethiopia. In this study we tried to assess the current status of HTPs in the study area. It might also have an implication in improving child health care practice and in reducing child morbidity and mortality.

## 2. Materials and Methods

Community based cross-sectional study was conducted in Axum Town, North Ethiopia, in 2013. The sample population was all mothers who have children less than 5 years old. The town is divided into 4 kebeles and kebeles also subdivided into ketenas (districts). A total of 9 ketenas, 3 ketenas from kebelle hawelti, and 2 ketenas from each of the rest of kebeles were selected. Afterwards, proportional allocation of samples systematic sampling method was used to select 752 mothers. Structured questionnaire adapted from the follow-up survey of HTPs in Ethiopia by EGLDAM in 2008 was used. Data was collected from mothers who have children less than 5 years old using interviewer administered questionnaire. Correction was done based on the feedback from the pretest. Data was checked and cleaned daily for completeness and consistency during data collection. Data was entered and analyzed using SPSS software (version 18.0) and descriptive statistics was calculated. It was coded and cleaned before analysis. The proposal was submitted to College of Health Sciences, Department of Nursing and Midwifery Institutional Review Board (IRB), Addis Ababa University for approval. Following approval, official letter of cooperation was written to Axum Town Administration Office from the Department of Nursing and Midwifery of Addis Ababa University. After getting permission from Axum Town Administration, data collectors were trained about the study. Study participants were informed OF the purpose, advantage, and disadvantage of the study, with the right to refuse at any stage of the interview. Confidentiality was assured for all the information provided and informed verbal consent was obtained prior to interview.

## 3. Results

### 3.1. Sociodemographic Characteristics

In this study a total of 752 mothers who had children less than five years old were interviewed with a response rate of 100%. The number of female children was 381 (50.7%) and mean age of children was 26.28 months (SD = +15.98; range: 1–59 months) while mean age of mothers was 30.55 years (SD = +6.22; range: 19–51 years). Majority of the respondents, 611 (81.2%), were Orthodox and 141 (18.8%) were Muslim. Regarding occupational status, about 588 (78.2%) of respondents had no work and 6 (0.8%) had other works like local clothes makers, Tella makers, and beauty salon workers. 379 (50.4%) attended primary school, 196 (26.1%) attended secondary school, and 100 (13.3%) were illiterate (Table 1).

### 3.2. Magnitude of Harmful Traditional Practices

Out of the 752 respondents 746 (99.2%) had information on at least one harmful traditional practice and 301 (40%) of them had information on all of the mainly recognized HTPs (uvula cutting, milk teeth extraction, FGM, and eyebrow incision), 588 (78.2%) knew about uvula cutting, and 493 (65.5%) knew about female genital mutilation. Three hundred seventy-two (49.5%) mothers mentioned family members as source of information and there was also another source mentioned by 4 (0.5%) respondents which was school. Most of mothers, 618 (82.2%), participating in this study had HTPs performed on themselves and of them 599 (79.6%) mothers reported that uvula cutting was performed on them. The mean age of children to perform HTPs was 4.61 weeks (SD = +10.5; range: 1–112). All of the HTPs, 660 (100%) cases, performed by traditional healers at their home. Minor complications like difficulty of swallowing, bleeding, swelling, and signs of infection happened in only in 80 (10.6%) cases after the procedure (Table 2).

**Figure 1:**
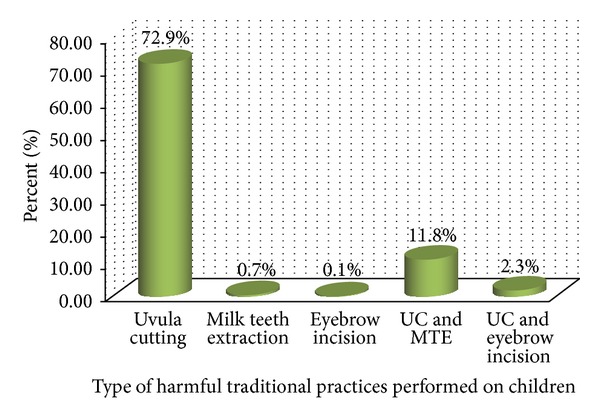
Types of harmful traditional practices among children less than 5 years in Axum Town, North Ethiopia, 2013.

From the total number of participants, 660 (87.8%) had performed at least one HTPs on their children. Among the HTPs performed on children, uvula cutting alone was practiced on 548 (72.8%) children and uvula cutting with milk teeth extraction as well as with eyebrow incision was performed on 89 (11.8%) and 17 (2.3%) children, respectively (Figure 1).

### 3.3. Reasons to Perform Harmful Traditional Practices

The main reasons to perform uvula cutting mentioned by mothers were to prevent swelling, pus, and rapture of the uvula which can lead the child to death as mentioned by 515 (68.5%) mothers. Having no better medical cure and its ability to prevent sore throat were mentioned by 96 (12.8%) and 97 (12.9%) respondents, respectively. Out of the respondents who practice milk teeth extraction, 62 (8.2%) reason out to prevent diarrhea and vomiting, prevention of teething problem as well as cure or prevention diseases were other reasons. All mothers who practice eyebrow incision, 18 (2.4%) explain only one reason to perform eyebrow incision which was to treat eye diseases (Table 3).

## 4. Discussion

The purpose of this study was to assess the magnitude of harmful traditional practices among children less than 5 years old in Axum Town, North Ethiopia. Almost all (99.2%) study participants had information about HTPs. This was higher than the study conducted in SNNPR which showed that a little higher number to half of the respondents (65.3%) had information about HTPs [11]. About 78.2% of mothers had information about uvula cutting; this was higher than the follow-up survey conducted in 2008 by EGLDAM which was 73.5% nationally but almost inline in Tigray Region 79.8% [4]. This difference might be because of time gap; the study area as well as sample size was smaller than the survey.

This study showed that uvula cutting was practiced on 87% of children which was lower than a study conducted in Dembia district, Northwest Ethiopia, in which uvula cutting was practiced by 99.5% of respondents [1]. But this was higher than the follow-up survey conducted in 2008 by EGLDAM in Ethiopia in which the prevalence of uvula cutting was 66.4% in Tigray Region [4]. The variation might be because of time gap. The second common HTP in this study was milk teeth extraction which was performed on 12.5% of children but it was much lower than a study conducted in Dembia district, Northwest Ethiopia, in 2001 (95.6%) [1]. In addition to that it was also much lower than the follow-up survey conducted in 2008 by EGLDAM in Ethiopia that reported prevalence of milk teeth extraction as 26.6% [4]. This might be because awareness of families of milk teeth extraction has been improved but not for uvula cutting because of the easily accessible medical service. The third common HTP identified was eyebrow incision which was performed on 2.4% of children, but it was almost null as compared to the study conducted in Dembia district, Northwest Ethiopia (82%) [1]. It might be due to the fact that fear of HIV/AIDS transmission as well as awareness of mothers of modern medicine to treat eye infections or diseases has been improved. In this study there was no FGM practiced on children. This was in line with the study conducted in Dembia district, in which the practice of female circumcision was limited to only some areas and was supported by a small number of people. But, according to the study, in SNNPR prevalence of FGM was 33% [11]. In Tigray Region as well, the prevalence of FGM was 21.2% according to the follow-up survey conducted in 2008 by EGLDAM [4]. The difference might be because awareness of families of the complications of FGM was improved by health education by health personnel and mass media.

The reason for performing uvula cutting mentioned by most (68.5%) mothers was to prevent swelling, pus, and rupture of the uvula which can lead the child to death. This was much higher than the same reason (21.7%) reported in the follow-up survey conducted in Ethiopia by EGLDAM in 2008 [4]. But, contrary to this study, a study conducted in Nigeria in 2011 suggested that the majority of patients (65.5%) did not know the indication for uvula cutting being performed on them [12]. This variation might be due to difference of cultural diversity of the respondents. Out of the respondents who practice milk teeth extraction (12.5%) the reasons mentioned were to prevent diarrhea and vomiting (8.2%), to prevent teething problem (1.1%), and to cure or prevent diseases. This was in line with the reasons mentioned in the follow-up survey conducted in Ethiopia by EGLDAM in 2008 [4]. This study was also in line with study of HTPs in SNNPR, in 2005 [11]. In this study eyebrow incision was performed to treat eye disease as mentioned by all mothers who performed it. This was also similar to the reasons listed in the follow-up survey conducted in 2008 by EGLDAM [4].

## 5. Conclusion

In the study we tried to assess magnitude of harmful traditional practices among children less than five years old in Axum Town, North Ethiopia. Participants of this study were mothers who had children less than five years old. Based on this study the following was concluded.

- Almost all of the respondents had information about the commonly recognized HTPs which were uvula cutting, milk teeth extraction, FGM, and eyebrow incision; family members were the main source of information.
- Majority of the mothers had uvula cutting, milk teeth extraction, and eyebrow incision performed on them.
- The common HTPs performed on children in this study were uvula cutting, MTE, and eyebrow incision. Uvula cutting was practiced alone as well as together with MTE and eyebrow incision.
- As mentioned by mothers the main reason of uvula cutting was to prevent swelling, pus, and rupture of the uvula which can lead the child to death.
- Prevention of diarrhea, vomiting, and teething problem was the reason described by mothers for performing milk teeth extraction.

## Figures and Tables

**Table 1:** Sociodemographic characteristics of children less than five years old in Axum Town, North Ethiopia, 2013.

Variable	Frequency (*n* = 742)	Percent
Sex		
Male	371	49.3
Female	381	50.7
Age of child in months		
0–4	47	6.2
5–9	92	12.2
10–14	107	14.2
15–19	51	6.8
20–24	87	11.6
25–29	40	5.3
30–34	62	8.2
35+	266	35.4
Age of mothers		
15–19	6	0.8
20–24	115	15.3
25–29	222	29.5
30–34	168	22.3
35–39	159	21.1
40–44	71	9.4
45+	11	1.5
Religion		
Orthodox	611	81.2
Muslim	141	18.8
Occupation		
Jobless	588	78.2%
Civil servant	56	7.4
Merchant	98	13.0
Farmer	4	0.5
Others∗	6	0.8
Ethnic group		
Tigraway	748	99.5
Amhara	4	0.5
Educational status		
Illiterate	100	13.3
Religious	17	2.3
Primary school	379	50.4
Secondary school	196	26.1
Higher education	60	8.0

*Others = local clothes makers, beauty salon workers, and local drinks makers.

**Table 2:** Harmful traditional practices among children less than five years old in Axum Town, North Ethiopia, 2013.

Variable	Frequency (*n*)	Percent (%)
Information about HTPs		
Yes	746	99.2
No	6	0.8
Information by type of HTPs∗		
Uvula cutting	588	78.2
Female genital mutilation (FGM)	493	65.5
Milk teeth extraction (MTE)	380	50.5
Eyebrow incision	362	48.1
Bloodletting	308	40.9
Source of information∗		
Mass media	314	41.7
Health personnel	410	54.5
Family members	372	49.5
Meeting	127	16.9
Others∗∗	4	0.5
Any HTPs performed on mother		
Yes	618	82.2
No	134	17.8
Type of HTPs performed on mother∗		
Uvula cutting	599	79.6
Female genital mutilation	5	0.7
Milk teeth extraction	43	5.7
Eyebrow incision	116	15.4
Bloodletting	16	2.13
HTPs performed on children		
Yes	660	87.8
No	92	12.2
In how many children		
All children	209	27.8
Only one child	158	21.0
Two children	175	23.3
Three and above	118	15.7
Minor problems happened after HTPs were performed		
Yes	80	10.6
No	580	77.1
Which problems happened after HTPs were performed		
Bleeding	16	2.1
Swelling	8	1.1
Difficulty of swallowing	45	6.0
Wound or infection	11	1.5

*More than one answer was given; ∗∗others = school.

**Table 3:** Reasons associated with harmful traditional practices among children less the 5 years old in Axum Town, North Ethiopia, 2013.

Variable	Frequency (*n* = 742)	Percent (%)
Reasons for performing uvula cutting∗		
To prevent swelling, pus, and rupture of uvula	515	68.5
No better cure in modern medicine	96	12.8
To prevent sore throat	97	12.9
To prevent vomiting	2	0.3
Reasons for performing MTE∗		
Prevents diarrhea and vomiting	62	8.2
Prevents problems of growth development	5	0.7
Root of teeth grows worms	21	2.8
MTE prevents or cures disease	9	1.2
Prevents teething problems	8	1.1
Reason for performing eyebrow incision		
Treatment of eye disease	18	2.4
Thinking if HTPs are harmful		
Yes	484	64.4
No	268	35.6
Which HTPs are harmful∗		
FGM	472	62.7
Eye borrow incision	338	44.9
bloodletting	336	44.7
Milk teeth extraction	315	41.9
Uvula cutting	168	22.3

*More than one response was given.
